# Thoracic endovascular aortic repair for type B aortic dissection with aberrant right subclavian artery: a single-center retrospective study

**DOI:** 10.3389/fcvm.2023.1277286

**Published:** 2023-12-07

**Authors:** Yanzhang Zeng, Ping Yuan, Qiang He

**Affiliations:** ^1^ Department of Vascular and Thyroid Surgery, Guizhou Provincial People’s Hospital, Guiyang, China; ^2^ Department of Intervention, Guizhou Provincial People’s Hospital, Guiyang, China

**Keywords:** type B aortic dissection, aberrant right subclavian artery, thoracic aortic endovascular aortic repair, retrospective study, outcome analysis

## Abstract

**Objective:**

To evaluate the outcomes of thoracic endovascular aortic repair (TEVAR) for type B aortic dissection (TBAD) with aberrant right subclavian artery (ARSA).

**Methods:**

A retrospective analysis was conducted on patients with TBAD and ARSA who underwent TEVAR between the period of January 2017 and December 2022. Patient demographics, computed tomography angiography (CTA) measurements, surgical procedures, and postoperative outcomes were reviewed.

**Results:**

A total of 9 patients (6 males and 3 females) were included in the study. 4 ARSA were reconstructed, 3 by periscope technique and 1 by *in vitro* fenestration technique. 3 left subclavian arteries (LSA) were reconstructed, 1 by the chimney technique and 2 by the single-branched stent technique. 2 patients underwent reconstruction of both ARSA and LSA. The overall technical success rate was 100%, with no occurrences of stroke, paraplegia, or mortality within 30 days. 1 patient experienced immediate type Ia endoleak, which resolved after 3 months. 1 patient developed weakness in the right upper limb, while 1 patient presented mild subclavian steal syndrome (SSS); both cases showed recovery during follow-up. The average follow-up duration was 35.6 ± 11.1 months, during which no reinterventions, deaths, or strokes were observed.

**Conclusion:**

Our limited experience involving 9 patients demonstrates that early and mid-term outcomes of TEVAR for the treatment of TBAD with ARSA are satisfactory.

## Introduction

The aberrant right subclavian artery (ARSA) is the most commonly observed variant of the aortic arch and its branches, originating from the aortic arch or descending aorta after the left subclavian artery (LSA). The reported incidence of ARSA ranges from 0.3% to 3% (1). In most cases, ARSA is asymptomatic, with only approximately 5% of patients experiencing symptoms such as dysphagia, dyspnea, cough, and other respiratory difficulties due to compression of the esophagus and trachea (2). In recent years, thoracic endovascular aortic repair (TEVAR) has emerged as the primary treatment for type B aortic dissection (TBAD) due to its minimal invasiveness and rapid recovery (3).

However, managing TBAD with ARSA poses challenges in TEVAR. Currently, there are no established guidelines or consensus on the management of TBAD with ARSA, and most studies available are limited to case reports. The decision to reconstruct the LSA and ARSA should take into account the proximal landing zone (PLZ), patency of the cerebral arterial circle (CAC), and dominance of the vertebral artery. In this retrospective study, we analyzed patients diagnosed with TBAD and ARSA who underwent endovascular repair to provide valuable insights for clinical treatment.

## Material and methods

### Patients

A retrospective analysis was conducted on patients diagnosed with TBAD and ARSA who underwent TEVAR at authors' institution between from January 2017 to December 2022. The study was approved by the Ethics Committee of Guizhou Provincial People's Hospital and the informed consents were obtained from all patients. The inclusion criteria were as follows: (1) Patients diagnosed with TBAD and ARSA; (2) Patients who underwent TEVAR with or without superior-arch artery revascularization. The exclusion criteria were as follows: (1) Presence of aortic connective tissue diseases, such as Marfan syndrome; (2) Prior history of aortic surgery; (3) Thoracic aortic aneurysm, penetrating aortic ulcers, or intramural hematoma. All patients underwent computed tomographic angiography (CTA) of the entire aorta with a slice thickness of 1 mm to assess the anatomical parameters of the aortic arch. Cerebral and cervical CTA were also performed to evaluate the CAC and identify the dominant vertebral artery. The reconstruction of the LSA and ARSA was determined based on the combination of CAC, dominant vertebral artery (DVA), and PLZ. If the CAC was patent, only the subclavian artery on the side of the DVA was reconstructed. However, in cases where the CAC was obstructed, simultaneous reconstruction of both LSA and ARSA was recommended to mitigate the risk of short-term or delayed cerebrovascular events (as shown in Table 1).

**Table 1:** Treatment options based on circle of willis and proximal landing zone.

CAC	PLZ > 15 mm			PLZ < 15 mm		
	LVAD	RVAD	E	LVAD	RVAD	E
Fluent	AC	AR	AR	LR + AC	LC + AR	LR + AR
Obstructed	AR	AR	AR	LR + AR	LR + AR	LR + AR

PLZ, proximal landing zone; CAC, cerebral arterial circle; AC, ARSA covered; LC, LSA covered; AR, ARSA reconstruction; LR, LSA reconstruction; LVAD, LVA dominant; RVAD, RVA dominant; E, equal dominant.

The indications for TEVAR included refractory hypertension, persistent chest or back pain, aortic diameter >55 mm and suspected or existing aortic rupture. The acute phase was defined as onset time of 14 days or less, the subacute phase as lasting between 15 and 90 days, and the chronic phase as exceeding 90 days (4). During the acute phase, conservative treatment was implemented by controlling blood pressure and heart rate. TEVAR was conducted during the subsequent subacute phase, except in cases requiring emergency interventions, such as rupture or impending rupture (persistent decrease in blood pressure or hemoglobin, suspected hemorrhagic pleural effusion), malperfusion syndrome, and progression of aortic dissection. The classification of TBAD was based on the location of the primary tear and the extent of involvement in the distal lesion (5).

### TEVAR procedures

All TEVAR procedures were performed under general anesthesia. The aortic stent grafts used in the study were C-Tag (Gore, Delaware, USA), Castor (Microport Medical, Shanghai, China), and Ankura (Lifetech, Shenzhen, China), as presented in Table 2. The criteria for selecting the correct stent graft size allowed for an oversize of 5%–10%. The femoral artery was surgically exposed or two Perclose Proglide vascular staplers (Abbott, Chicago, United States) were pre-set. The SELDINGER technique was employed for puncturing. The stent graft was advanced using a super stiff guidewire (Lunderquist, COOK, Bloomington, USA). For the reconstruction of the ARSA or LSA, Fluency (Bard, New Jersey, USA) and Viabahn (Gore, Delaware, USA) stents were utilized. In the periscope technique, the guide wire was pre-set in ARSA, and both the stent graft and periscope stent were released simultaneously. A guide wire was inserted from the 8F sheath of the left brachial artery into the femoral artery, followed by the insertion of Castor's 4F catheter through the guide wire into the left brachial artery. Subsequently, the Castor stent guide wire was inserted into this catheter. The Castor stent graft and wire were then simultaneously moved upward, and the stent was released once it reached the planned position. During the fenestration, the anterior segment of the Castor stent was partially exposed and fenestrated *in vitro*. A short 5F sheath was placed through the right radial artery, and the guide wire from this sheath was passed through the fenestration of the Castor stent via ARSA, into the femoral artery, and retrieved from the sheath. Finally, a Viabahn stent was inserted into ARSA via the femoral artery.

**Table 2:** Procedures and outcomes.

Patient	Sex/age	Max diameter,mm	Classiffication	PLZ, mm	CAC	DVA	Endovascular procedures	Brand of the stent graft	Outcomes	Follow-up period, months
1	M/60	60	B3,6	10	F	R	LC + AP	Gore C-Tag		48
2	M/46	42	B3,9	16	F	L	AC	Lifetech Ankura		60
3	M/59	33	B3,9	11	F	E	LB + AP	Microport Castor		39
4	F/64	39	B3.5	6	O	L	LB + AF	Microport Castor		15
5	M/48	45	B4,9	25	F	L	AC	Gore C-Tag	weakness of the right upper limb, relieved after 5 months	51
6	M/41	37	B3,5	19	F	L	AC	Gore C-Tag	SSS, relieved after medical treatment	33
7	M/72	30	B3,9	8	F	L	LCH + AC	Gore C-Tag	Type Ia endoleak, disappeared at 3 months	28
8	M/50	44	B3,10	20	F	L	AC	Lifetech Ankura		26
9	F/55	29	B4,5	22	F	R	AP	Gore C-Tag	Lung cancer	20

PLZ, proximal landing zone; CAC, cerebral arterial circle; DVA, dominant vertebral artery; F, fluent; O, obstructed; LCH, LSA chimney; AP, ARSA periscope; LBS, LSA branched stent; AF, ARSA fenestration; AC, ARSA covered; LC, LSA covered; L, Left; R, Right; SSS, subclavian steal syndrome.

### Medical treatment and follow up

**Figure 1:**
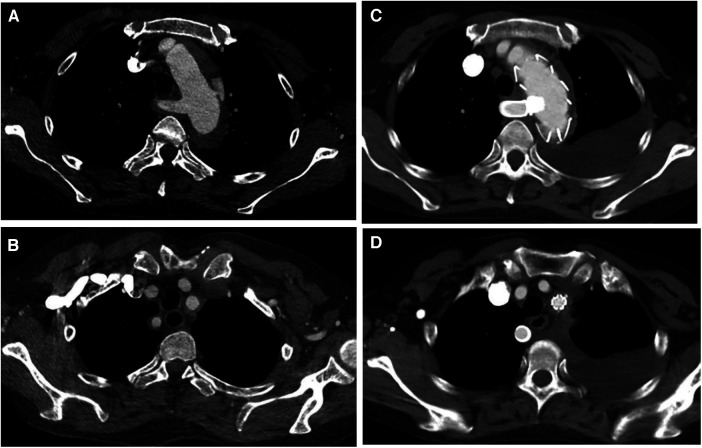
(**A**,**B**) Preoperative CT angiography of patient 4, who underwent castor stent graft and fenestration; (**C**,**D**) postoperative CT angiography, blood flow was fluent in LSA and ARSA.

Discharged patients were instructed to diligently monitor and regulate their blood pressure. For patients who underwent ARSA or LSA reconstruction, a daily oral dose of 100 mg aspirin was administered. The occurrence of stroke, spinal cord ischemia, endoleak, subclavian steal syndrome (SSS), and reinterventions were considered as early and late morbidity and were included in the follow-up assessments. Follow-up appointments were scheduled for discharged patients at 1, 6, and 12 months after surgery, and annually thereafter. The end point of the follow-up period was June 2023, and data collection was conducted through outpatient medical records, CTA images, and telephone calls. The preoperative and postoperative CTA images are shown in Figure 1.

## Results

A total of 9 patients, including 6 men and 3 women, were included in this study. The mean age range of patients was 55 ± 9.8 years (range 41–72). None of the patients had prior knowledge of ARSA before admission. Among the patients, 5 were in the acute stage of aortic dissection, 1 was in the subacute stage, and 3 were in the chronic stage. 1 patient experienced TBAD as a result of iatrogenic injury during coronary angiography. Further details regarding the characteristics of the patients can be found in Table 3.

**Table 3:** General and mophological characteristics of the patients.

Features	*M* or *x *± *s*	Percent or range
Age, years	55.0 ± 9.8	41–72
Sex, Male	6	66.7%
Hypertension	8	88.9%
Coronary artery disease	2	22.2%
Diabetes	1	11.1%
Chronic obstructive pulmonary disease	3	33.3%
Pleural effusion	2	22.2%
CT measurements		
Aortic diameter at the distal edge of LSA, mm	30.6 ± 1.9	28–34
Length from LSA to primary entry tear, mm	14.9 ± 6.9	6–25
Max diameter of descending aorta, mm	39.9 ± 9.5	29–60
Kommerell diverticulum	1	11.1%
Dissection of ARSR ostium	2	22.2%
Diameter of ARSR, mm	10.3 ± 1.4	9–13
Follow-up time	35.6 ± 11.1	15–60

**Figure 2:**
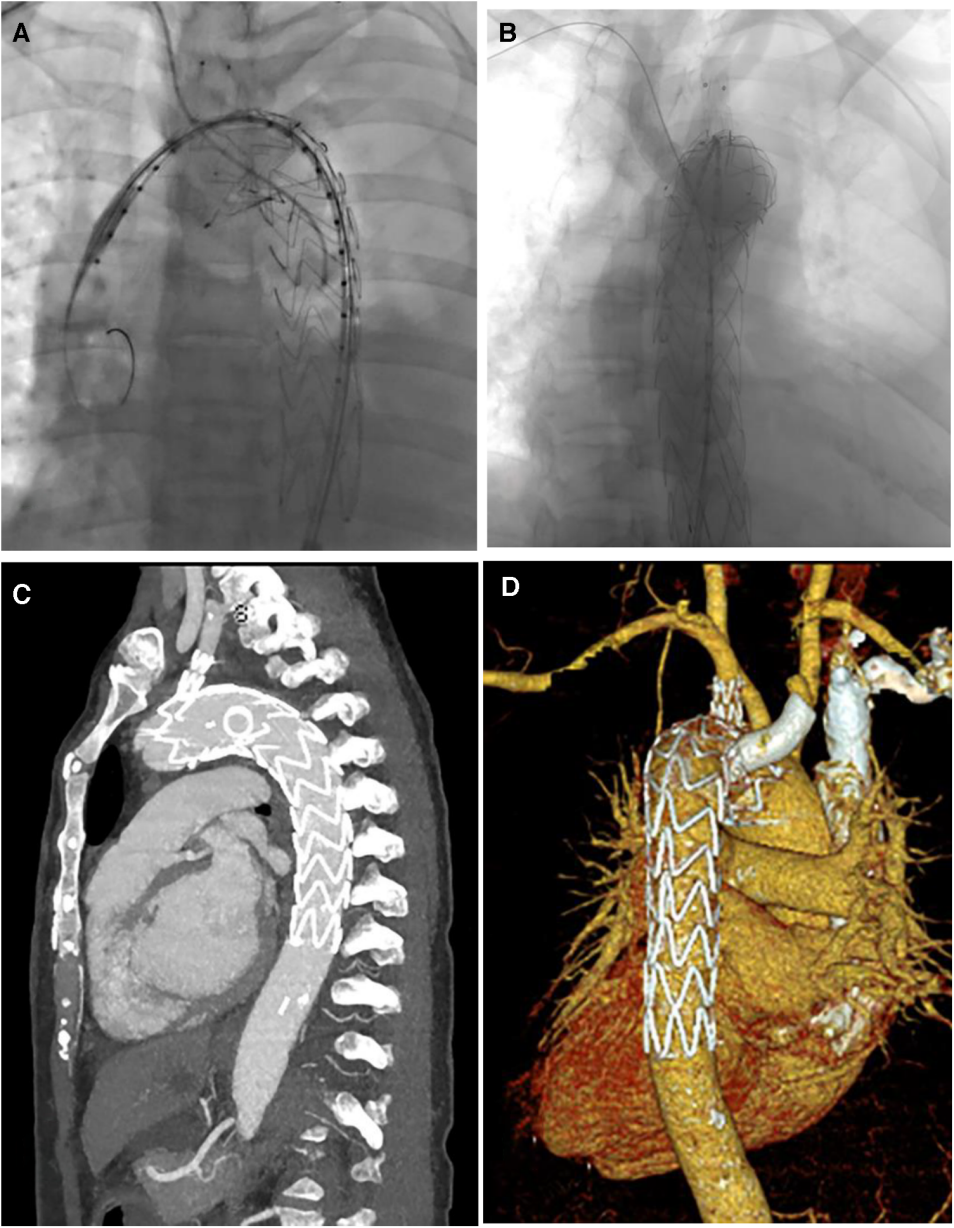
(**A**,**B**) Digital subtraction angiography in TEVAR of patient 4; (**C**,**D**) CT image remodeling 1 month after TEVAR.

Among the patients, 4 had a PLZ measuring less than 15 mm. Coverage of 1 LSA was conducted, while 2 LSA were reconstructed using single-branched stents, and 1 LSA was reconstructed by chimney technique. 5 ARSA were covered. 4 ARSA reconstructions were performed, 3 of which were performed by periscope technique and 1 by fenestration *in vitro* (as shown in Figure 2). The technical success rate achieved 100%, and there were no cases of stroke, spinal cord ischemia, death within 30 days following surgery. No patients necessitated ongoing care in the intensive care unit following the surgical procedure. Detailed information on the lesions and surgical procedures performed can be found in Table 2.

During follow-up, 1 patient has a slight type Ia endoleak and it disappeared 3 months later. No patients underwent reintervention and no stent occlusion, infection, or migration occured. After ARSA coverage, 1 patient developed mild SSS, confirmed by Doppler ultrasound, and manifested as mild vertigo. Significant alleviation of the vertigo was observed after medical treatment 5 months post-surgery. Another patient experienced weakness in the right upper limb, which was relieved through functional exercise. No stroke or death occurred during follow-up. 1 patient was diagnosed with lung cancer 12 months after surgery.

## Discussion

ARSA is a common congenital variation in the aorta. In most cases, patients have no obvious clinical symptoms and do not require treatment (1, 2). TBAD is a clinical acute aortic syndrome with a high mortality rate. Due to its reduced trauma and faster recovery, TEVAR has gradually replaced open surgery as the preferred treatment option for TBAD (6). However, TBAD with ARSA poses additional challenges in TEVAR. Currently, there are no published treatment guidelines specifically addressing this combination. Open and hybrid procedures have been employed for the treatment of TBAD with ARSA. Di Marco et al. (7) and Abuharb et al. (8) reported the use of Frozen Elephant Trunk in patient diagnosed with aortic dissection and ARSA. The prognosis is favorable but the surgical trauma is extensive. Chen et al. (9) and Chien et al. (10) reported cases of TBAD with ARSA which are treated by hybrid technique. In a study conducted Ding et al. (11), the overall technical success rate and prognosis were satisfactory, despite a reported 12.5% incidence of brachial plexus injury among patients. Although hybrid surgery has reduced trauma compared to open surgery, TEVAR still offers significant advantages in terms of intensive care unit utilization, postoperative recovery time, and surgical trauma. With the development of interventional technology, total endovascular treatment is increasingly applicated to the treatment of TBAD with ARSA.

Currently, there remains a scarcity of guidelines or expert consensus regarding the criteria for ARSA reconstruction. Since the ARSA ostium is distal to the LSA, the stent graft will inevitably cover it. Regular TEVAR procedures that involve sacrificing the LSA are associated with a notable rise in neurological complications, including cerebral infarction and spinal cord ischemia (12, 13). In the general population, the left DVA appears more frequently compared to the right DVA. Consequently, sacrificing only the ARSA reduces the risk of neurological complications compared to sacrificing the LSA. If the length of PLZ is less than 15 mm, LSA reconstruction is necessary to achieve sufficient length, and clinicians must decide whether to reconstruct ARSA. Zhou et al. (14) reported on 9 patients who had a covered ARSA, and there were no incidents of severe stroke or spinal cord ischemia after the surgery. Zhang et al. (15) combined the proximal landing zone with the side of the DVA to determine whether both subclavian arteries should be preserved. However, when the CAC is occluded, reconstruction of the DVA alone is insufficient to completely prevent stroke. As individuals age and atherosclerosis advances, it may result in stenosis or occlusion of the CAC. In our study, 2 patients with CAC occlusion, as indicated by CTA, underwent reconstruction of both ARSA and LSA without experiencing any neurological complications. With the advancement of endovascular reconstruction of supra-arch branch vessels, the LSA and ARSA should be reconstructed as much as possible in the future, given the experience and resources available.

To date, there is a lack of published literature that compares various methods of ARSA reconstruction. Parallel stents are commonly used for reconstruction of the LSA or ARSA (16). To our knowledge, published literatures about reconstruction of ARSA with periscope are mainly case reports (17, 18). In our study, 3 ARSA were reconstructed by periscope technique and no endoleak was observed. Fenestration, being a more recent technique compared to parallel stents, has had fewer reported cases in TBAD with ARSA. Gafoor et al. (19) reported a case involving a patient with a thoracic aortic aneurysm and ARSA, who underwent TEVAR with a customized, *in situ* fenestrated stent graft, based on CT image remodeling. Xu et al. (20) conducted a study on the endovascular repair of type B aortic intramural hematoma with ARSA. 8 patients received treatment using handmade fenestration stents, yielding positive outcomes. In our study, we performed *in vitro* fenestration for ARSA reconstruction in 1 patient, and during the follow-up period, no endoleak or neurological events were observed. Single-branched stents can be used for ARSA reconstruction as well. Zhang et al. (21) reported the application of an embedded modular single-branched stent for endovascular repair of TBAD with ARSA; however, this device has not seen widespread adoption. In recent years, a single-branched stent graft named “Castor”, specifically designed for TBAD involving LSA, has been widely used and has achieved satisfactory results (22, 23). Pang et al. (24) reconstructed 5 ARSAs during TEVAR using Castor stents and the single branch was directly used to reconstruct the ARSA. However, this treatment option is limited by the anatomical condition of the lesion. We suggest that he periscope technique may be a suitable method for ARSA reconstruction due to three reasons. Firstly, unlike chimney technique that may result in type Ia endoleak, the periscope technique has minimal impact on the proximal part of the stent graft. Although the periscope technique may promote the occurrence of type Ib endoleak, its incidence and prognosis are better than those of type Ia endoleak. Secondly, The periscope technique's procedures are easy to perform, and the devices are also easy to obtain. Finally, compared to fenestration technique, the periscope technique has the advantages of maintaining stent stability. Furthermore, *in vitro* fenestration technique requires caution due to the risk of inaccurate fenestration positioning. With the increasing availability of fenestration methods, we consider that mechanical in-situ fenestration can be a promising reconstruction method. The single-branched stent such as Castor presents as a reliable option for reconstruction when the LSA is the DVA, effectively reducing the incidence of type I endoleaks. In addition, in cases requiring reconstruction of the ARSA, the risk of severe endoleaks from the use of two parallel stents can be minimized by employing a single-branched stent.

The present study has several limitations. Firstly, the study is retrospective and based on a small sample size of only 9 cases, resulting in a low level of evidence. Hence, further prospective studies with larger samples are warranted. Secondly, due to the limited sample size, it was not feasible to compare the prognosis among different ARSA and LSA reconstruction modalities. Lastly, the study is lacking long-term follow-up results.

## Conclusion

Our limited experience involving only 9 patients suggest that TEVAR is a feasible treatment option for TBAD with ARSA, demonstrating satisfactory early and mid-term outcomes. However, further researches with larger sample size are required.

## Data Availability

The raw data supporting the conclusions of this article will be made available by the authors, without undue reservation.
